# Combined detrusor-trigone BTX-A injections for urinary incontinence secondary to neurogenic detrusor overactivity

**DOI:** 10.1038/sc.2015.143

**Published:** 2015-08-11

**Authors:** C Hui, X Keji, J Chonghe, T Ping, O Rubiao, Z Jianweng, D Xiangrong, Z Liling, H Maping, L Qingqing, L Qiuling, H Jiebing, H Tanghai

**Affiliations:** 1 Department of Urology, Guangdong Provincial Work Injury Rehabilitation Hospital, Jinan University, Guangzhou, China; 2 Department of Urology, Guangzhou First Municipal People's Hospital, Guangzhou, China; 3 Department of Urology, Qingyan City People's Hospital, Jinan University, Guangdong, China

## Abstract

**Objectives:**

The objective of this study was to evaluate the effect and safety of trigonal injection of botulinum toxin A (BTX-A) for patients with neurological detrusor overactivity (NDO) with incontinence.

**Methods:**

A prospective, multicenter, single-blind and randomized controlled trial was conducted between June 2011 and June 2014. Spinal cord injury patients with urinary incontinence secondary to NDO overactivity were recruited. At a 1:1 ratio, patients randomly received 200 U BTX-A intradetrusor injections excluding the trigone (the control group) or 160 U intradetrusor and 40 U intratrigonal injections (the experimental group). Patients were evaluated at baseline and at 4 and 12 weeks after injection. The efficacy and safety outcomes included Incontinence-Specific-Quality-of-Life Instrument (I-QoL), voiding volume, urinary incontinence episodes, complete dryness, maximum detrusor pressure (*P*_detmax_) and volume at first involuntary detrusor contraction (*V*_FIDC_). Vesicoureteral reflux (VUR) and other adverse events were recorded.

**Results:**

Ninety-six patients were recruited and 91 of them completed the trial. Among the 91 patients, 47 were randomized to the experimental group and 44 to the control group. There were no significant differences in baseline evaluation items (gender, age, duration of spinal cord injury, level of neurological injury, AIS (the American Social Injury Association) scores) between the two groups. At 12 weeks, the improvement was significantly better in the experimental group compared with that in the control group for I-QoL (26.01 vs 18.75, *P*=0.01), mean urinary incontinence episodes (−5.22 vs −4.68 per day, *P*=0.01), complete dryness (13 vs 5, *P*=0.03), mean voiding volume (159.72 vs 139.07 ml, *P*=0.02), *P*_detmax_ (−33.34 vs −28.02 cmH_2_O, *P*=0.04) and VFIDC (106.81 vs 97.86 ml, *P*=0.02), duration of first detrusor contraction (−41.54 vs −18.65 s, *P*=0.03) and the number of patients with detrusor contraction (−20 vs −9, *P*=0.02). In both the groups, no patients developed VUR.

**Conclusions:**

BTX-A intradetrusor and intratrigonal injections are more effective compared with those excluding the trigone for patients with NDO with incontinence. Intratrigonal injections do not induce VUR.

## Introduction

Detrusor overactivity (DO) is characterized by spontaneous or provoked involuntary detrusor contractions during storage phase in urodynamic investigation.^1, 2^ Neurogenic DO (NDO) is DO caused by various neurogenic diseases such as brain tumors, dementia, multiple sclerosis, Parkinson's disease, stroke and spinal cord injury (SCI).^3^ NDO can cause a variety of long-term complications such as urinary incontinence, stones, hydronephrosis, recurrent urinary tract infection and VUR; the most dangerous being damage of renal function. These complications may markedly impact the quality of life of people with SCI, including limiting their behavior, causing social embarrassment and possibly threatening their life.^4^

Botulinum toxin A (Botox; Allergan) is an acetylcholine release inhibitor and a neuromuscular blocking agent indicated by the beneficial treatment effect on NDO patients who have an inadequate response to or are intolerant to anticholinergic medication according to both clinical and urodynamic test, such as improvement percentage of Incontinence-Specific-Quality-of-Life Instrument (I-QoL), reduction of urinary incontinence episodes and lower detrusor pressure and so on.^5, 6^

In the past 10 years, intradetrusor injection of BTX-A was performed while avoiding the trigone to prevent VUR.^7^ However, several studies reported that there are abundant sensory nerve fibers in bladder trigone, and its smooth muscles are sensitive to small pressure changes.^8, 9^ Based on these findings, combined detrusor-trigone BTX-A injections may desensitize the bladder and thereby help to reduce bladder uninhibited contraction and dyssynergia.

To our knowledge, several studies reported satisfactory clinical results about combined detrusor-trigone BTX-A injections.^10, 11, 12, 13, 14, 15^ However, most of these studies were small and single-center experience. Therefore, the objective of this prospective, multicenter, single-blind and randomized controlled trial was to evaluate the efficacy and safety of combined detrusor-trigone BTX-A injections for NDO with urinary incontinence.

## Materials and methods

### Study population

This trial was conducted in three different institutions from 18 June 2011 to 23 June 2014. Eligible SCI in-patients for the study were recruited. Inclusion criteria were: (1) at least 18 years old with various neurogenic disorders; (2) urodynamic DO with urinary incontinence; (3) an inadequate response or intolerance to oral anticholinergic drugs; (4) participants or their caregiver could perform clean intermittent catheterization (CIC). Exclusion criteria were: (1) an allergy to BTX-A; (2) women were pregnant, lactating or planning to become pregnant during the course of the trial; (3) acute urinary tract infection. All patients were given a thorough explanation of both modes of treatment and provided written informed consent before injection. A urology resident not participating in the operations assigned patients to treatment based on a randomization schedule from a random-number table balanced in blocks of 6. The study was approved by the ethics committees of the three participating hospitals.

### Study design

**Figure 1:**
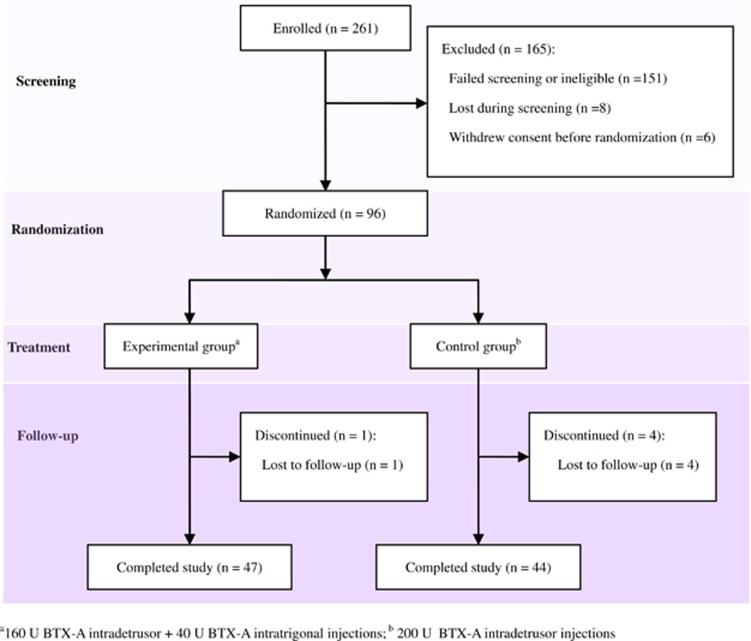
Flow chart shows screening, randomization, treatment and follow-up of the study. A full color version of this figure is available at the *Spinal Cord* journal online.

The study was a prospective, multicenter, single-blind and randomized comparison of combined detrusor-trigone BTX-A (Botox; commercial lot 2024; Allergan; 100 U ml^−1^) injections with detrusor BTX-A injections (Figure 1). At a 1:1 ratio, patients were randomly assigned to an experimental group, who received 160 U intradetrusor plus 40 U intratrigonal injections, or to a control group, who received 200 U BTX-A intradetrusor injections excluding the trigone.

### Study outcomes

The primary end-point outcomes were the changes in the videourodynamic test evaluated at baseline, and at 12 weeks after injection: (1) incidence of VUR; (2) maximum detrusor pressure during first involuntary detrusor contraction (*P*_detmaxIDC_) during filling storage; (3) volume at first involuntary detrusor contraction (*V*_FIDC_); (4) duration of first DO; and (5) incidence of DO.

Secondary end-point outcomes assessed at baseline and during weeks 4 and 12 after injections were I-QoL,^16^ voiding volume, urinary incontinence episodes between CICs per 24 h and complete dryness. The I-QoL contains 22 items evaluating problems related to incontinence. Items are scored on a 5-point scale with values ranging from 1 (extreme) to 5 (not at all). Scores were then converted to a scale ranging from 0 (worst I-QoL) to 100 (best I-QoL). Voiding volume is defined as voided volume by CIC plus spontaneous voids. Complete dryness is defined as <1 incontinence episode per 24 h. All these outcomes were determined from seven consecutive days of the patient's bladder diary. The related adverse events were recorded throughout the study.

### Injections

**Figure 2:**
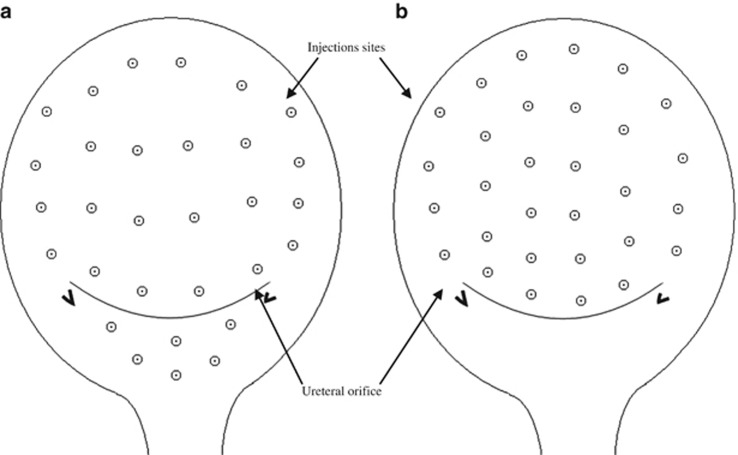
Location of BTX-A injection sites. (**a**) The 160 U intradetrusor plus 40 U intratrigonal and (**b**) 200 U intradetrusor injections.

Injections were performed with no anesthesia or under epidural anesthesia in the operating room with a 21F rigid cystoscope (Ackermann, Schaffhausen, Switzerland). The bladder was instilled with 100–150 ml sterile saline to achieve adequate visualization so as to avoiding the blood vessels during injections. A 23-gauge needle (Cook Urological Incorporated, Bloomington, IN, USA) was inserted ~2 mm into the detrusor. The 200 U Botox vials (100 U each) were reconstituted in a total of 30 ml sterile saline (6.7 U ml^−1^). A total of 30 injections of 1 ml each were administered, evenly distributed about 1 cm apart across the bladder wall. Patients in the experimental group had six 1-ml (total 40 U) injections into the bladder trigone sparing a 5 mm distance to the vicinity of the ureteral orifices and the bladder neck, and 24 1-ml injections (total 160 U) into the bladder wall (Figure 2a). Patients in the control group had 1-ml injections at 30 sites into the bladder wall, avoiding the trigone. Both procedures were performed by a single senior urologist with extensive experience in BTX-A injections (Figure 2b). In both procedures, a 16 Foley catheter had been inserted for 3–5 days. Oral prophylactic antibiotics (except aminoglycosides) were administered on the day of treatment.

### Statistical analysis

Student's *t*-test was used for comparison of related variables of both groups and results are presented as means±s.d. The *χ*^2^ test was used for categorical data. A *P-*value ⩽0.05 was considered statistically significant. Statistical analyses were performed with SPSS 13.0 software (SPSS Inc., Chicago, IL, USA).

## Results

Of the 261 participants screened at three participating hospital, 165 patients were excluded from the study, consisting of 151 who failed to screening because of SCI patients with age below 18 years old (*n*=27), detrusor underactivity or acontractility (*n*=78), acute urinary tract infection or epididymitis (*n*=28), an allergy to BTX-A (*n*=2), compromised respiratory function (*n*=16); another eight who lost during screening (*n*=8); and six who withdrew consent before randomization (*n*= 6). Among 96 who underwent randomization, 5 patients were lost to follow-up at week 4 because of moving abroad (*n* =1) or losing contact (*n*=4). Therefore, a total of 91 SCI patients (47 assigned to the experimental group and 44 assigned to the control group) completed 12 weeks of follow-up and data were available and analyzed (Figure 1). At baseline, there were no significant differences between groups with respect to any demographic or baseline characteristics (Table 1).

### Efficacy

#### The primary end-point outcomes

No patient developed unilateral or bilateral VUR at week 12. Patients with 160 U BTX-A intradetrusor plus 40 U BTX-A intratrigonal injections had statistically greater improvement compared with those in the control group for *P*_detmaxIDC_ (−33.34 vs −28.02 cmH_2_O, *P*=0.04), VFIDC (106.81 vs 97.86 ml, *P*= 0.02), duration of first detrusor contraction (−41.54 vs −18.65 s, *P*=0.03) and the number of patients with detrusor contraction (−20 vs −9, *P*=0.02) at week 12 (Table 2).

#### Secondary end-point outcomes

Table 3 shows that significant changes were already present in both groups by week 4 for mean dairy UI episodes (−4.74 vs −4.25 per day, *P*=0.03), complete dryness (13 vs 5, *P*=0.03), mean voiding volume (159.39 vs 140.16 ml, *P*=0.04) and I-QoL (24.97 vs 19.59, *P*=0.02), respectively. Furthermore, patients in the experimental group still yielded significant improvements in mean urinary incontinence episodes (−5.22 vs −4.68 per day, *P*=0.01), complete dryness (13 vs 5, *P*=0.03), mean voiding volume (159.72 vs 139.07 ml, *P*=0.02) and I-QoL (26.01 vs 18.75, *P*=0.01) compared with those in the control group at week 12.

#### Safety and tolerability

During the first week after injection, we observed three patients (two in the experimental group, one in the control group) with mild transient hematuria. Two patients in the control group reported bladder discomfort at week 1. All patients did not require any medication or surgical intervention.

## Discussion

Since the introduction of botulinum toxin for the treatment of NDO, the majority of urologists have avoided trigone infiltration to prevent VUR.^5, 7^ Our study demonstrates that it does not induce vesicoureteral reflux during the 12-week follow-up after injection. In 2007, Karsenty *et al.*^10^ firstly reported that in the 11 female patients with nonneurogenic overactive bladder who received 100 U botox injection (five sites) into trigone, there was no induced VUR and the patient who had VUR at baseline showed no change in VUR grade at the 6-week videourodynamic test. In 2008, Citeri *et al.*^11^ retrieved 240 patients with NDO who were treated with 250 U Dysport into the trigone, no VUR was recorded. In 2010, Pinto *et al.*^12^ and Abdel-Meguid^13^ reported that 26 patients with bladder pain syndrome/interstitial cystitis and nine patients with NDO who received 100 U botox injections into 10 trigonal sites, respectively, and no cases of VUR occurred in both studies. In 2011, Kuo^14^ and Manecksha *et al.*^15^ also reported no incidences of VUR in 68 patients and 11 patients with idiopathic DO after injections of 100 U botox injections into the bladder trigone.

The primary aim in the treatment of NDO is to ensure that the detrusor pressure remains within safe limits during both the filling phase and the voiding phase for protection of the upper urinary tract.^17^ The present trial reports significant improvements in these parameters were evident with the 200 U dose of BTX-A injection with or without trigone injection. In this trial, although the follow-up period was relatively short, BTX-A intradetrusor and intratrigonal injections were superior to those excluding the trigone with respect to NDO. Specifically, we found (1) according to our trial that only 57.45% of patients with NDO observed involuntary detrusor contraction at week 12; (2) the patients in the experimental group showed significant reductions in duration of the first detrusor contraction; and (3) most importantly, the *P*_detmax_ decreased more significantly of including rather than excluding the trigone to levels traditionally considered safe for the upper urinary tract when bladder trigone was included rather than excluded. In our study, there was some differences in the total intradetrusor injected dose and number of injected sites between the experimental group (160 U, 24 sites) and the control group (200 U, 30 sites). However, Schurch *et al.*^7^ compared 300 U (30 sites) and 200 U (20 sites) BTX-A intradetrusor injections for NDO and found no significant differences in the safety and efficacy for the two different doses and sites. In addition, another study comparing 100 U vs 150 U of BTX-A in patients with idiopathic DO and also found that the clinical effect was equivalent in terms of symptom reduction and quality of life improvement.^18^ Moreover, the bladder trigone muscles are sensitive to minute pressure changes and have an important role in initiating involuntary detrusor contractions, which spread throughout the bladder muscles.^9^ Hence, trigonal denervation may help decrease such involuntary contractions, and thus we did not increase the intradetrusor injections dose in the experiment group.

Another important aims in the treatment of NDO is to improve the patient's QoL.^17^ The improvements in urodynamic outcomes also transfer into the increases in scores of I-QoL in both groups. Significant benefits were evident by week 12. However, the mean change of I-QoL in the experiment group was substantially higher compared with that in the control group at week 12 (26.01 vs 18.75, *P*=0.01). The larger improvement in I-QoL in the experimental group may be related to the following changes: (1) the proportional reduction of daily urinary incontinence episodes was significantly larger with BTX-A intradetrusor plus intratrigonal injections compared with those excluding the trigone (71.95 vs 68.50%, *P*=0.02); (2) the patients in the experimental group showed greater improvement in voiding volume compared with those in the control group (159.72 vs 139.07 ml, *P*=0.02); and (3) most importantly, 13 patients with BTX-A intradetrusor plus intratrigonal injections developed complete dryness postoperatively, and their I-QoL was very high. Thus, these patients are less likely to worry about the disturbance from urinary incontinence, which affects their physical activities, social relationships and feelings.

No patients developed systemic or significant adverse events of treatment in this trial. Similar safe outcomes have also been demonstrated by previous studies at 1.5–3 months.^10, 11, 12, 13, 14, 15^

A limitation of this study is that the number of patients was relatively fewer. Therefore, further studies are warranted.

## Conclusions

Our results have demonstrated that BTX-A intradetrusor plus intratrigonal injections are more effective compared with those excluding the trigone for NDO of SCI patients. Intratrigonal injections do not induce VUR at 12 weeks postinjection.

## DATA ARCHIVING

There were no data to deposit.

## Figures and Tables

**Table 1:** Baseline characteristics of the participants

*Characteristic*	*Experimental group* *(*n=*47)*	*Control group* *(*n=*44)*	P-*value*
Age (years), mean (s.d.)	29.83 (11.77)	28.46 (10.36)	0.56
Gender, men, *n* (%)	28 (59.57)	23 (52.27)	0.48
Weight (kg), mean (s.d.)	67.16 (19.25)	65.93 (20.62)	0.77
Duration of spinal cord injury (months), mean (s.d.)	20.04±6.56	19.43 ±5.48	0.63
Episodes^a^ of urinary incontinence (n/d), mean (s.d.)	7.38±2.64	7.34±2.31	0.94
Level of SCI injury, C6–C8/ T1–T12/ L1–L5, *n*	6/28/13	9/26/9	0.52
AIS grade, A/B/C, *n*	27/12/8	29/10/5	0.66
Prior anticholinergic drugs use, *n* (%)	43 (91.49)	41 (95.35)	0.76
Prior CIC use, *n* (%)	45 (100)	42 (100)	0.95

Abbreviations: AIS, the American Social Injury Association; CIC, clean intermittent catheterization; SCI, spinal cord injury.

a The variable was assessed from the patients' 7-day bladder diary.

**Table 2:** Mean baseline and change from baseline in primary outcomes: VUR, *P*_detmax_, *V*_FIDC_, duration of first detrusor contraction and patients with detrusor contraction

*Outcome*	*Experimental group* *(*n=*47)*	*Control group* *(*n=*44)*	P-*value*
*Incidence of VUR,* n *(%)*			
Baseline	0	0	NS
Week 12	0	0	NS
			
*Pdetmax^a^* *(cmH_2_O), mean (s.d.)*			
Baseline	68.38±17.96	68.94±16.01	0.87
Week 12	−33.34 ±20.27	−28.02 ±15.18	0.04
			
*VFIDC*^a^ *(ml), mean (s.d.)*			
Baseline	165.55±41.96	170.98±36.48	0.51
Week 12	106.81±56.15	97.86±42.79	0.02
			
*Duration of first detrusor contraction*^a^ *(s), mean (s.d.)*			
Baseline	87.22±67.43	84.61±69.87	0.86
Week 12	−41.54±38.27	−18.65±49.13	0.03
			
*Number of patients with involuntary detrusor contraction,* n *(%)*			
Baseline	47 (100)	44 (100)	NS
Week 12	−20 (−42.55)	−9 (−20.45)	0.02

Abbreviations: NS, no significance; *P*_detmax_, maximum detrusor pressure during first involuntary detrusor contraction; *V*_FIDC_, volume at first involuntary detrusor contraction; VUR, vesicoureteral reflux.

^a^Only includes patients who had an DO.

**Table 3:** Mean baseline and change from baseline in secondary outcomes: I-QoL, voiding volume^a^, urinary incontinence episodes^a^ and patients with complete dryness^a^

*Outcome*	*Experimental group* *(n=47)*	*Control group* *(n=44)*	P-v*alue*
*Urinary incontinence episodes (*n *per day), mean (s.d.)*			
Baseline	7.38±2.64	7.34±2.31	0.94
Week 4	−4.74±0.95	−4.25±1.18	0.03
Week 12	−5.22±0.91	−4.68±1.06	0.01
			
*Patients with complete dryness,* n *(%)*			
Baseline	0	0	NS
Week 4	13 (27.66)	5 (11.36)	0.03
Week 12	13 (27.66)	5 (11.36)	0.03
			
*Voiding volume (ml), mean (s.d.)*			
Baseline	202.55±48.67	218.95±53.35	0.13
Week 4	159.39±41.08	140.16±48.79	0.04
Week 12	159.72 ±39.11	139.07 ±41.61	0.02
			
*I-QoL, mean (s.d.)*			
Baseline	37.27± 7.14	36.12±5.88	0.41
Week 4	24.97±9.32	19.59±11.83	0.02
Week 12	26.01±11.56	18.75±15.18	0.01

Abbreviations: I-QoL, Incontinence-Specific-Quality-of-Life Instrument; voiding volume, voided volume by CIC plus spontaneous voids; NS, no significance.

a These variables were assessed from the patients' 7-day bladder diary.
